# Association Between Delay to First Shock and Successful First-Shock Ventricular Fibrillation Termination in Patients With Witnessed Out-of-Hospital Cardiac Arrest

**DOI:** 10.1161/CIRCULATIONAHA.124.069834

**Published:** 2024-10-28

**Authors:** Remy Stieglis, Bas J. Verkaik, Hanno L. Tan, Rudolph W. Koster, Hans van Schuppen, Christian van der Werf

**Affiliations:** 1 Anesthesiology, Amsterdam UMC Location AMC, the Netherlands (R.S., H.v.S.).; 2 Quality of Care, Amsterdam Public Health, the Netherlands (R.S., H.v.S.).; 3 Department of Clinical and Experimental Cardiology, University of Amsterdam Heart Center, Amsterdam UMC, the Netherlands (B.J.V., H.L.T., R.W.K., C.v.d.W.).; 4 Heart Failure and Arrhythmias, Amsterdam Cardiovascular Sciences, the Netherlands (B.J.V., H.L.T., R.W.K., C.v.d.W.).; 5 Netherlands Heart Institute, Utrecht, the Netherlands (H.L.T.)

**Keywords:** cardiopulmonary resuscitation, defibrillators, electric countershock, emergency medical services, heart arrest, out-of-hospital cardiac arrest, ventricular fibrillation

## Abstract

**BACKGROUND:**

In patients with out-of-hospital cardiac arrest who present with an initial shockable rhythm, a longer delay to the first shock decreases the probability of survival, often attributed to cerebral damage. The mechanisms of this decreased survival have not yet been elucidated. Estimating the probability of successful defibrillation and other factors in relation to the time to first shock may guide prehospital care systems to implement policies that improve patient survival by decreasing time to first shock.

**METHODS:**

Patients with a witnessed out-of-hospital cardiac arrest and ventricular fibrillation (VF) as an initial rhythm were included using the prospective ARREST registry (Amsterdam Resuscitation Studies). Patient and resuscitation data, including time-synchronized automated external defibrillator and manual defibrillator data, were analyzed to determine VF termination at 5 seconds after the first shock. Delay to first shock was defined as the time from initial emergency call until the first shock by any defibrillator. Outcomes were the proportion of VF termination, return of organized rhythm, and survival to discharge, all in relation to the delay to first shock. A Poisson regression model with robust standard errors was used to estimate the association between delay to first shock and outcomes.

**RESULTS:**

Among 3723 patients, the proportion of VF termination declined from 93% when the delay to first shock was <6 minutes to 75% when that delay was >16 minutes (*P*_trend_<0.001). Every additional minute in VF from emergency call was associated with 6% higher probability of failure to terminate VF (adjusted relative risk, 1.06 [95% CI, 1.04–1.07]), 4% lower probability of return of organized rhythm (adjusted relative risk, 0.96 [95% CI, 0.95–0.98]), and 6% lower probability of surviving to discharge (adjusted relative risk, 0.94 [95% CI, 0.93–0.95]).

**CONCLUSIONS:**

Every minute of delay to first shock was associated with a significantly lower proportion of VF termination and return of organized rhythm. This may explain the worse outcomes in patients with a long delay to defibrillation. Reducing the time interval from emergency call to first shock to ≤6 minutes could be considered a key performance indicator of the chain of survival.

**Clinical Perspective**

**What Is New?**

- In this large, well-characterized cohort of patients with a witnessed out-of-hospital cardiac arrest and ventricular fibrillation as initial rhythm, >93% of the shocks successfully terminated ventricular fibrillation when the first shock was delivered within 6 minutes.
- Every minute of delay to first shock was associated with a 6% lower incidence of successful defibrillation.
- A shorter delay to first shock was also associated with a higher proportion of organized rhythm after the first shock, a lower total number of shocks, a higher proportion of transportation with sustained return of spontaneous circulation, and a higher chance of survival to discharge.

**What Are the Clinical Implications?**

- Reducing the time interval from emergency call to first shock to ≤6 minutes could be considered a key performance indicator of the chain of survival, and interventions to meet this criterion should be initiated.

In patients with out-of-hospital cardiac arrest (OHCA), ≈10% to 40% have an initial shockable rhythm.^1^ It has been well established that survival to hospital discharge is more likely when the event is witnessed by a bystander and early defibrillation is provided.^1^ In recent years, the availability and early use of automated external defibrillators (AEDs) has increased considerably, associated with higher survival rates.^2–5^

When initiation of ventricular fibrillation (VF) is witnessed by emergency medical services (EMS) or during continuous electrocardiographic monitoring in-hospital, prompt defibrillation usually leads to successful termination of VF.^6,7^ However, when the time between initiation of VF and the first shock increases, the likelihood of survival decreases.^8–10^

The association between duration of VF and the likelihood of terminating VF by the first shock has not been thoroughly studied in patients with OHCA. In particular, the degree to which the likelihood of successful defibrillation decreases when the duration of VF increases has not been quantified, nor has the possible additional benefit of early defibrillation to other important measures, such as return of organized rhythm, return of spontaneous circulation (ROSC) after a successful shock, or need for subsequent shocks.

Establishing this association could have a clinical impact. Estimating the duration of VF at which the likelihood of successful defibrillation decreases significantly is important to determine a target time to first shock. Such a key performance indicator is necessary to arrange the logistics of the chain of survival; for example, to decide where public AEDs should be placed.^11,12^

The aim of this observational study was to analyze the association between the delay to first shock and successful defibrillation and restoration of organized rhythm, based on data from the prospective ARREST registry (Amsterdam Resuscitation Studies) of OHCA.

## METHODS

### Setting and Study Population

ARREST is an ongoing prospective registry of all patients with OHCA in the province of North Holland in the Netherlands since 2005. The study region covers 2508 km^2^ and has a population of ≈2.6 million inhabitants. Between 2009 and 2018, the study area was extended with the eastern region of Twente, which covers 1504 km^2^ and has a population of ≈631 000 inhabitants. Both areas include a mix of rural and urban areas. For this study, the STROBE cohort (Strengthening the Reporting of Observational Studies in Epidemiology) reporting guidelines were used (Appendix in the Supplemental Material).^13^

In case of an OHCA, the emergency dispatch center provides cardiopulmonary resuscitation (CPR) instructions to the caller, activates the national volunteer responder system, and dispatches first responders (police and fire department) to start basic life support as soon as possible, including an AED.^14^ In the Netherlands, an AED is used before ambulance arrival in ±60% of OHCA.^15^ Two advanced life support–level ambulances are dispatched, staffed by an ambulance nurse and driver. Ambulance nurses are specialized nurses who provide advanced life support, including manual defibrillation, advanced airway management, intravenous medication, and mechanical chest compression. During the study period, most EMS services in the ARREST study region used Lifepak 15 manual defibrillators (Stryker) set to manual mode, which were set to charge to 200 J for the first shock and 360 J for all subsequent shocks, with electrodes in the standard anterolateral position. One EMS service used Corpuls^3^ defibrillators, which were set to charge to 200 J for all shocks, with electrodes in the standard anterolateral position.

The ARREST data collection and informed consent procedure were approved by the institutional review board of Amsterdam UMC, and details on the data collection have been reported previously.^16^ All patients who survived were asked for informed consent. Data from patients who did not survive could be used without informed consent. When consent was refused, an anonymous data set including general characteristics and defibrillator data was available for analysis.

For the current analysis, adult or pediatric patients with a witnessed OHCA and VF as the initial rhythm between 2011 and 2019 irrespective of the underlying cause were included. All unwitnessed arrests were excluded, as the time between loss of consciousness and first shock could not be estimated accurately. In addition, patients with an implantable cardioverter defibrillator were excluded.

The data set used for this study will not be made publicly available, but reasonable requests may be sent to the corresponding author for review.

### Data Collection and Definitions

Patient and resuscitation data were collected following the standard procedures of ARREST, according to the Utstein recommendations.^17^ The routine data collection of ARREST includes information from the dispatch centers, EMS, including the recordings from EMS defibrillators, AED data, and clinical data from treating hospitals up to discharge, as previously described.^4,16,18^ When a public, police, or firefighter AED was used, the AED ECG data were downloaded by study personnel, and the clock time of the AED recording was synchronized to correct for clock drift. EMS personnel transmitted the manual defibrillator data, which were time synchronized as well.

Residential areas were defined as private homes, including nursing homes and elderly homes. Degree of urbanization was defined as rural areas (<1000 addresses/km^2^) or urbanized areas (≥1000 addresses/km^2^). Time of day was categorized into night (12:00 am to 5:59 am), morning (6:00 am to 11:59 am), afternoon (12:00 pm to 5:59 pm), or evening (6:00 pm to 11:59 pm). First responders were defined as any dispatched assistance units, such as police, firefighter, or coast guard. Volunteer responders were defined as volunteer civilians (which may include off-duty medical personnel, police officers, and firefighters) dispatched by text-message system.^14^

### ECG Analysis

The initial rhythm was determined from the first ECG recording, either from an AED or EMS defibrillator. All first shocks were manually annotated in the ECGs and analyzed for shock success. A successful shock was defined as a shock that terminated VF for ≥5 seconds regardless of the subsequent rhythm. When the postshock rhythm was definitely nonshockable but the exact rhythm could not be determined (eg, because of CPR artefacts), the rhythm was classified as an unknown nonshockable rhythm.

In patients with an EMS-witnessed OHCA, delay to first shock was defined as the time interval between the initiation of VF and the first shock. In all other patients, delay to first shock was defined as the time interval between the emergency call to the dispatch center and the first shock by any defibrillator. When the first (AED) shock was delivered before the emergency call to the dispatch center, delay to first shock was considered to be <0 minutes.

### Outcomes

The primary outcome was termination of VF for ≥5 seconds after the first shock. Secondary outcomes were return of organized rhythm and asystole at 5 seconds after the first shock, ROSC before transport sustained until arrival at the emergency department, survival to discharge, and the total number of prehospital shocks, all in relation to the time from emergency call to first shock.

### Statistical Analysis

Categorical data were expressed as frequencies and percentages. Continuous data were presented as means (±SD) or medians with interquartile ranges (IQR). Differences between patients with a successful or failed termination of VF after the first shock were tested using the Pearson χ^2^ test or the Mann-Whitney *U* test when appropriate. The χ^2^ for trend test was used to evaluate trend differences.

To calculate the relative risk and adjusted relative risk to estimate change of successful VF termination 5 seconds after first shock, return of organized rhythm 5 seconds after first shock, multiple shocks delivered during the complete prehospital resuscitation, sustained ROSC before transport, and survival to hospital discharge in relation to the delay to first shock, a Poisson regression model with robust standard errors was used. The covariates sex, age, CPR before EMS arrival, rural or urbanized area, public or residential area, and time of day were added to the model to correct for possible confounding. Cases with missing data were omitted from the analyses.

*P*<0.05 was considered statistically significant. All statistical analyses were performed with SPSS v28 for Windows (IBM Corp.).

## RESULTS

**Figure 1.**
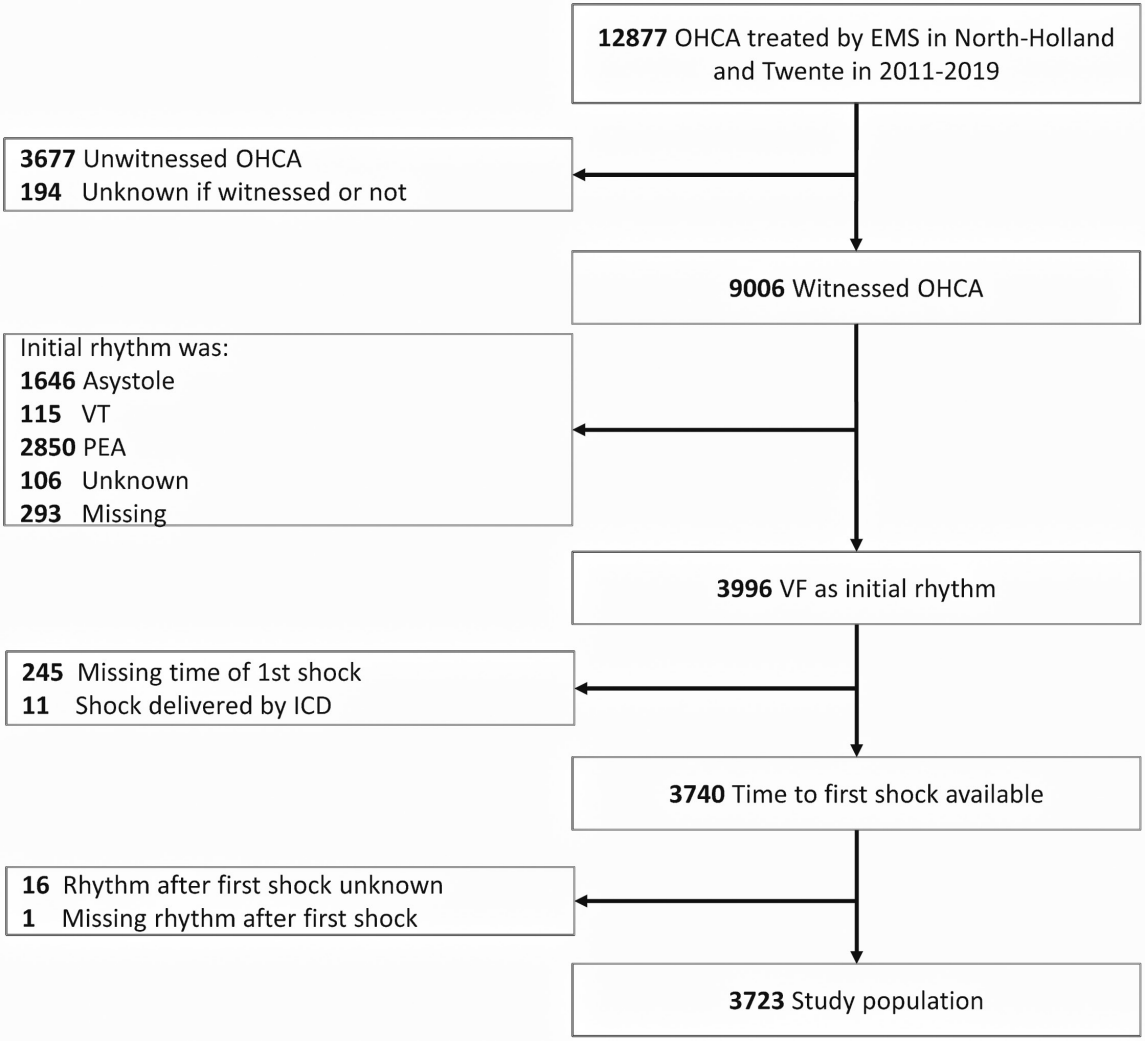
**Flowchart of the study population.** EMS indicates emergency medical services; ICD, implantable cardioverter defibrillator; OHCA, out-of-hospital cardiac arrest; PEA, pulseless electrical activity; VF, ventricular fibrillation; and VT, ventricular tachycardia.

A total of 3723 patients with a witnessed OHCA with VF as initial rhythm were analyzed (Figure 1), including 206 patients (6%) for whom EMS witnessed the cardiac arrest. Patient and resuscitation characteristics are detailed in Table 1. CPR was initiated by bystanders or first responders before EMS arrival in 3129 of 3715 patients (84%). In 2315 of 3723 patients (62%), the first shock was delivered by an AED, including 49 of 2315 patients (2%) for whom the first AED shock was delivered before the emergency call to the dispatch center.

**Table 1.**
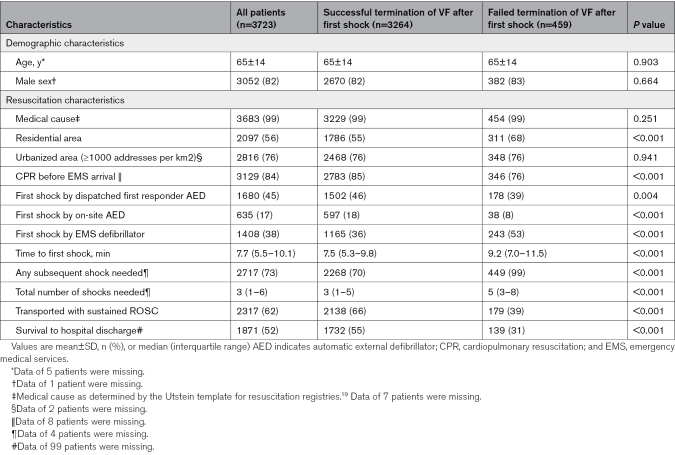
Patient and Resuscitation Characteristics

### Non–EMS-Witnessed Cases

#### Primary Outcome

**Figure 2.**
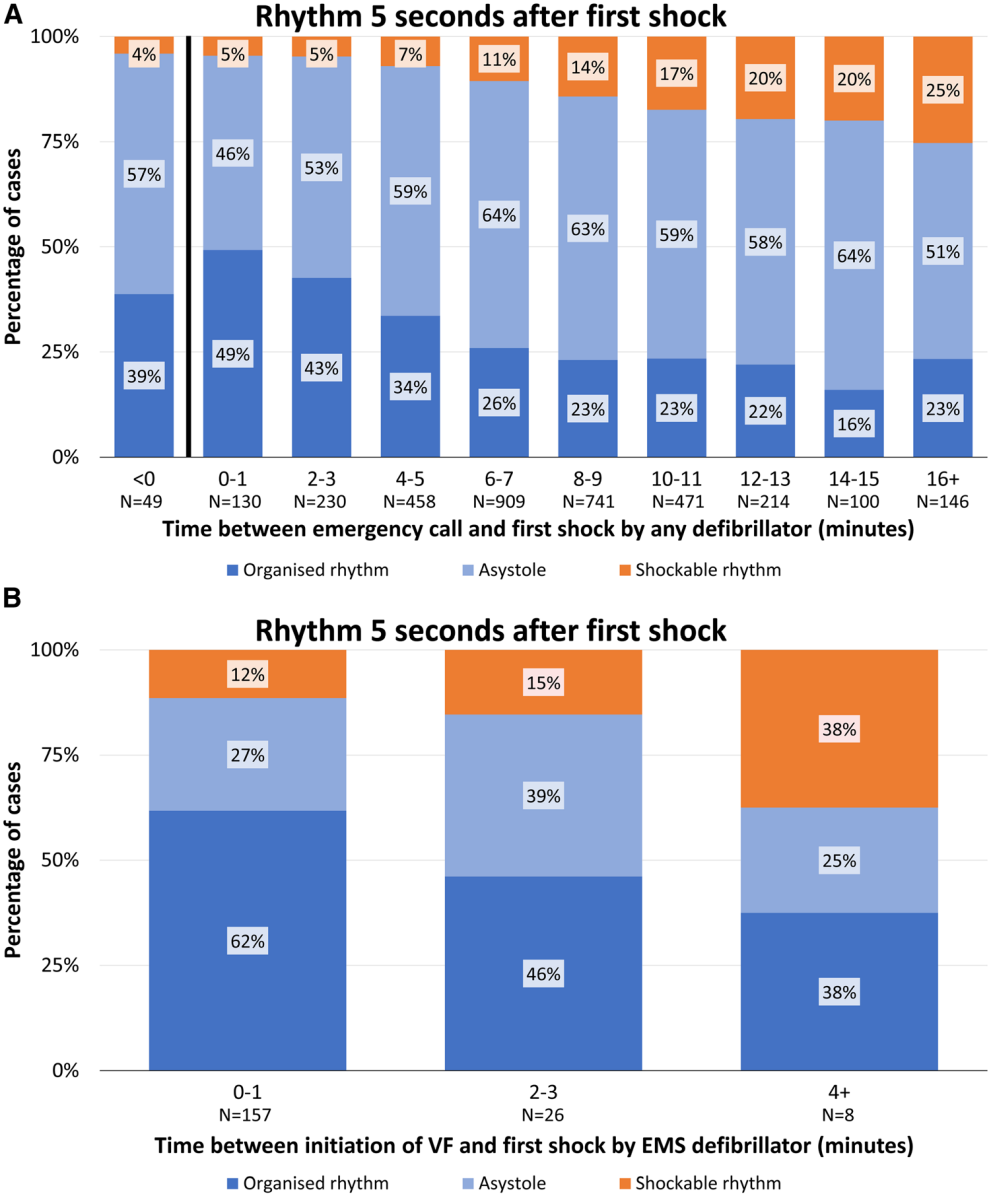
**Rhythm 5 seconds after the first shock in patients with a witnessed out-of-hospital cardiac arrest with ventricular fibrillation as initial rhythm. A**, Rhythm 5 seconds after the first shock in patients with a non–emergency medical services (EMS)–witnessed out-of-hospital cardiac arrest (OHCA) with ventricular fibrillation (VF) as initial rhythm. Rhythm 5 seconds after first shock in relation to the time interval between emergency call and first shock. The 49 patients within the <0 category are patients in whom an automated external defibrillator shock was delivered before the call to the dispatch center was received. χ^2^ for trend *P*<0.001. **B**, Rhythm 5 seconds after the first shock in patients with an EMS-witnessed OHCA with VF as initial rhythm. Rhythm 5 seconds after first shock in relation to the time interval between initiation of VF and first shock in patients with an EMS-witnessed OHCA. χ^2^ for trend *P*=0.007.

**Figure 3.**
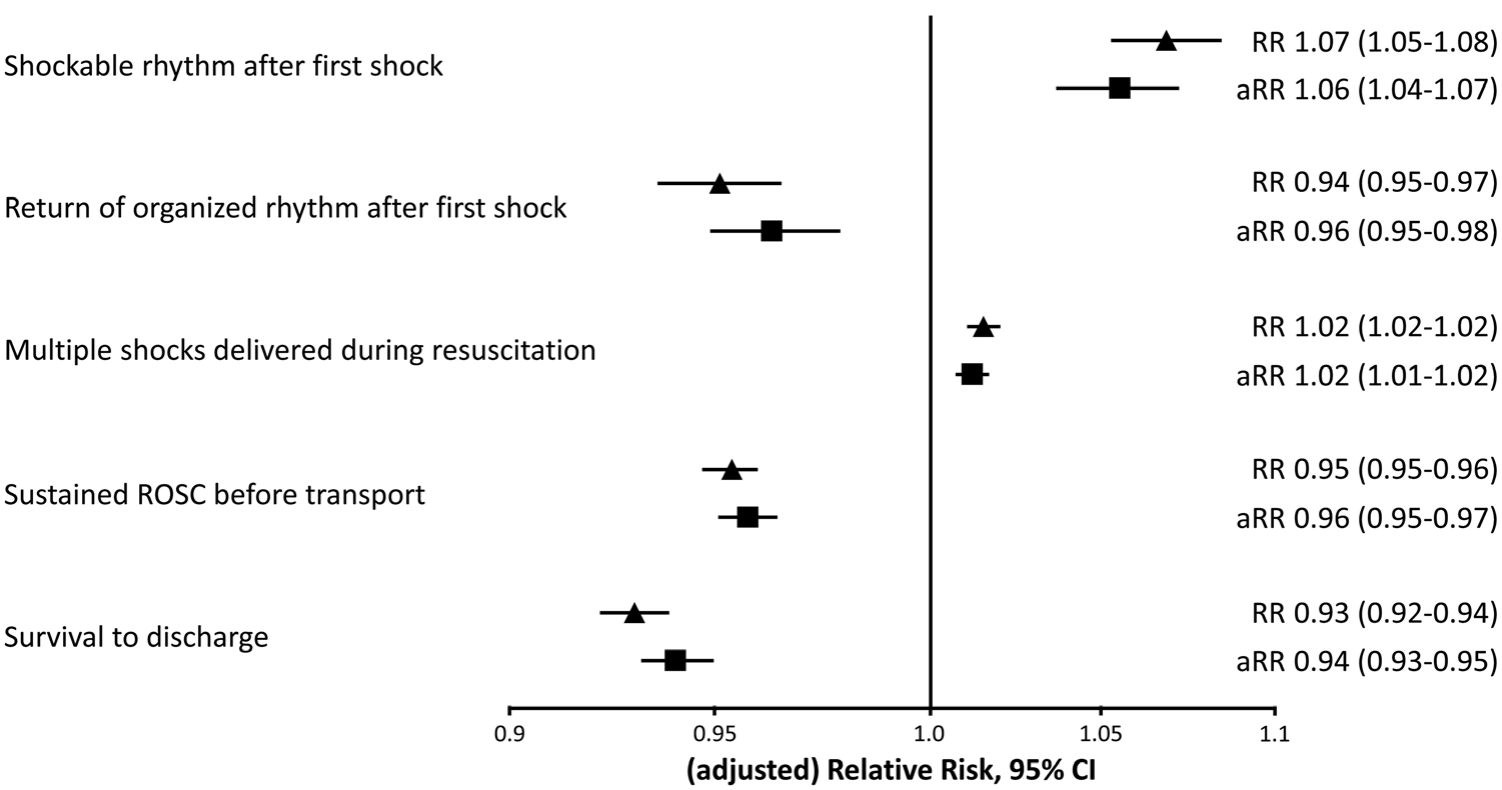
**Estimated adjusted relative risk in outcomes per additional minute of delay to first shock.** Estimated risk ratios (RRs) in outcomes per additional minute of delay to first shock compared with the reference category and adjusted for sex, age, rural or urban area, public or residential, and time of day using Poisson regression model with robust standard errors. Reference categories: nonshockable rhythm 5 seconds after first shock; no return of organized rhythm 5 seconds after first shock; 1 prehospital shock delivered during resuscitation; no sustained return of spontaneous circulation (ROSC) before transport; deceased before hospital discharge. aRR indicates adjusted relative risk.

Among the 3448 patients with an analyzable rhythm 5 seconds after shock not witnessed by EMS, VF was successfully terminated by the first shock in 3014 of 3448 (87%). VF was successfully terminated by the first shock in 47 of 49 patients (96%) in whom the first shock was delivered before the emergency call, and in 124 of 130 (95%) when the delay to first shock was <1 minute. First shock success declined gradually to 109 of 146 (75%) when delay to first shock was >16 minutes (*P*_trend_<0.001; Figure 2A). Every additional minute of delay from emergency call to first shock was associated with a statistically significant 6% higher probability of an unsuccessful shock (adjusted relative risk, 1.06 [95% CI, 1.04–1.07]; Figure 3).

#### Secondary Outcomes

In patients for whom the OHCA was not witnessed by EMS, an organized rhythm and asystole after delivery of the first shock occurred in 949 of 3448 (28%) and 2065 of 3448 (60%), respectively. The proportion of patients with an organized rhythm after the first shock decreased from 64 of 130 (49%) to 34 of 146 (23%) when delay from emergency call to first shock increased from <2 minutes to >16 minutes (*P*_trend_<0.001; Figure 2A), whereas the proportion of patients with asystole after the first shock did not change (60 of 130 [46%] to 75 of 146 [51%]; *P*_trend_=0.319; Figure 2A).

**Figure 4.**
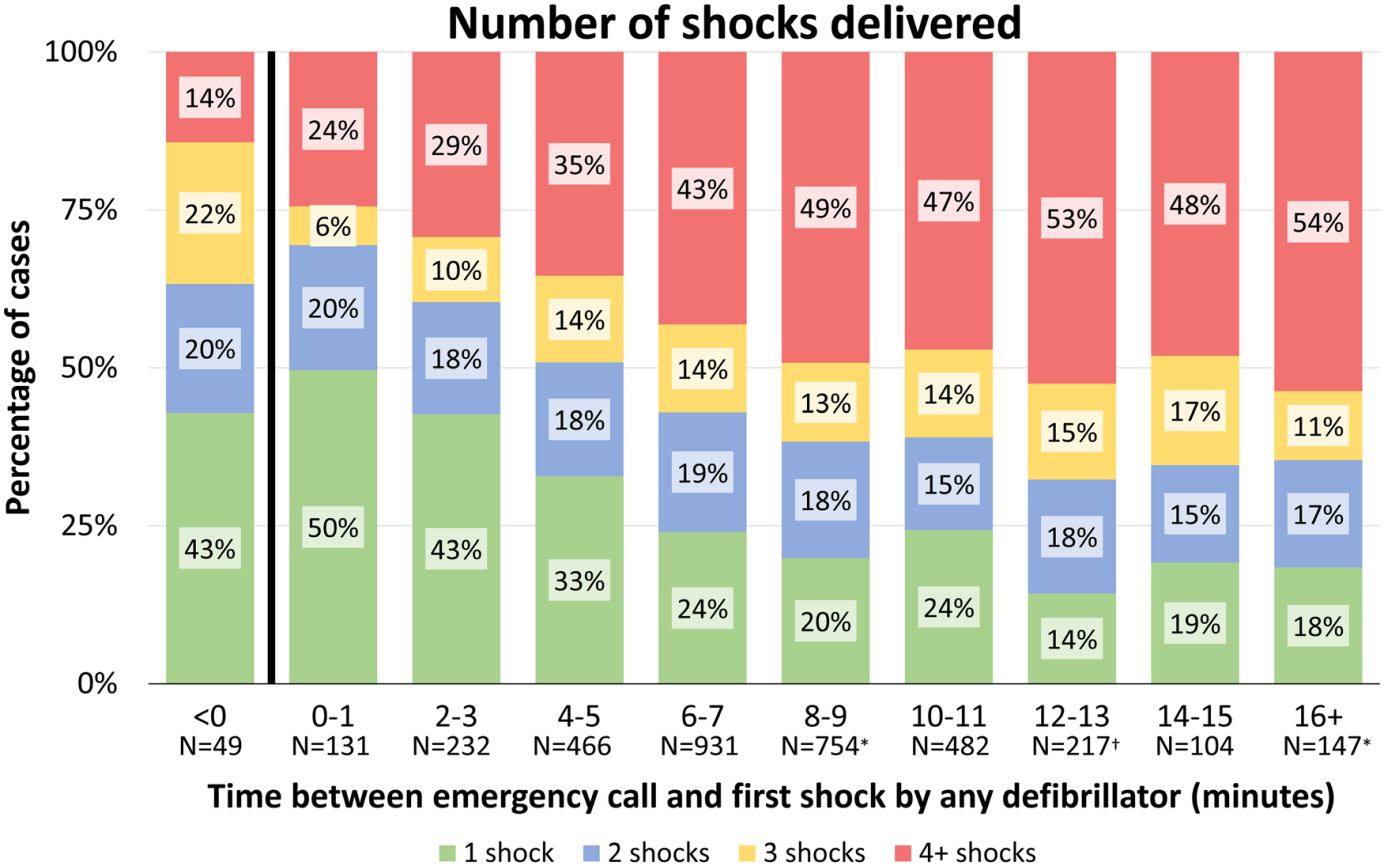
**Total number of prehospital shocks in patients with a non–emergency medical services–witnessed out-of-hospital cardiac arrest.** Total number of shocks delivered during the resuscitation attempt in the prehospital phase using any defibrillator (automated external defibrillator or emergency medical services defibrillator) in relation to the time interval between call to the dispatch center and first shock. The 49 patients within the <0 category are patients in whom an automated external defibrillator shock was delivered before the call to the dispatch center was received. χ^2^ for trend *P*<0.001. *Data of 1 patient were missing. †Data of 2 patients were missing.

In the total non–EMS-witnessed population, the median number of prehospital shocks was 3 (IQR, 1–6). The median number of shocks was higher in patients receiving the first shock >16 minutes after the emergency call than in those who received the first shock within 1 minute (4 [IQR, 2–7] versus 2 [IQR, 1–3], respectively; *P*_trend_<0.001; Figure 4). Patients who received the first shock before the emergency call received a median of 2 shocks (IQR, 1–3). Thus, a longer time interval between the emergency call and first shock was associated with a higher probability to receive multiple prehospital shocks (adjusted relative risk, 1.02 [95% CI, 1.01–1.02] per 1 minute of additional delay to first shock; Figure 3).

**Figure 5.**
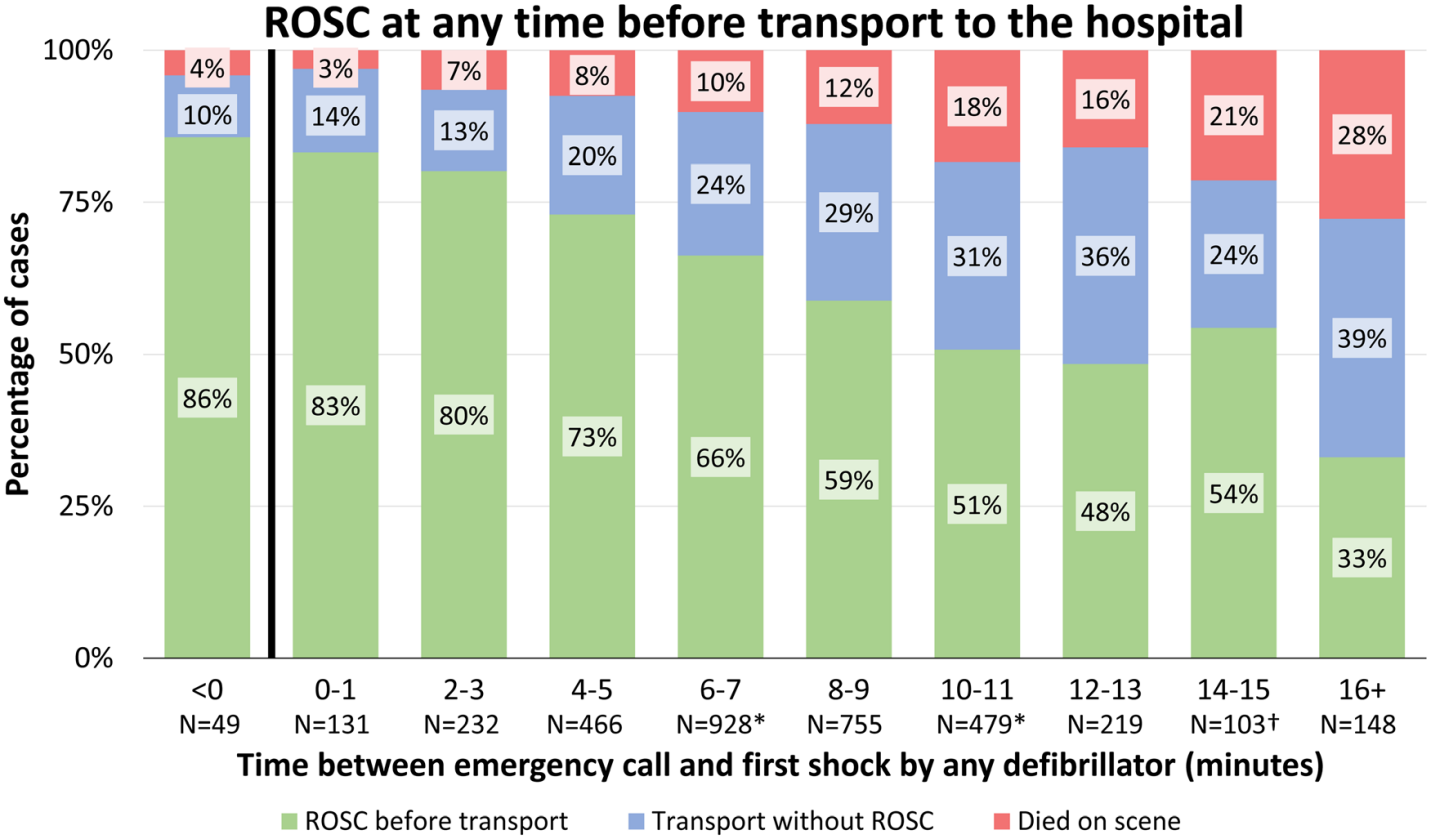
**Return of spontaneous circulation in relation to delay to first shock in patients with a non–emergency medical services–witnessed out-of-hospital cardiac arrest with ventricular fibrillation as initial rhythm.** Percentages of patients with return of spontaneous circulation (ROSC), patients who were transported to the hospital with ongoing cardiopulmonary resuscitation, or patients who died on scene in relation to delay to first shock. The 49 patients within the <0 category are patients in whom an automated external defibrillator shock was delivered before the call to the dispatch center was received. χ^2^ for trend P<0.001. *Data of 3 patients were missing. †Data of 1 patient were missing.

In 2189 of 3517 patients (62%), sustained ROSC was obtained before transport. This was observed most frequently in patients with the shortest delay to the first shock (Table 2; Figure 5). Of the total group of 3441 patients, 1730 (50%) survived to discharge, with survival rates being highest in patients with the shortest delay to first shock and decreasing with increasing delay to the first shock (Table 2).

**Table 2.**
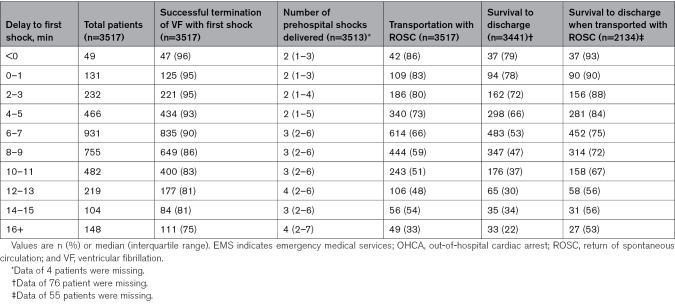
Delay to First Shock Related to Transportation With Total Number of Prehospital Shocks, Return of Spontaneous Circulation, and Survival to Discharge in Non–Emergency Medical Services–Witnessed Out-of-Hospital Cardiac Arrest

### EMS-Witnessed Cases

Among patients for whom initiation of VF was witnessed by EMS, VF was successfully terminated by the first shock in 139 of 157 (88%) when delay to first shock was <2 minutes, and in 22 of 26 (85%) when delay to first shock was 2 to 4 minutes (Figure 2B). The proportion of patients with an organized rhythm and asystole was 97 of 157 (62%) and 42 of 157 (27%), respectively, when delay to first shock was up to 2 minutes, and 12 of 26 (46%) and 10 of 26 (39%), respectively, when delay to first shock was 2 to 4 minutes (Figure 2B).

## DISCUSSION

In this large well-characterized cohort of patients with a witnessed OHCA, VF as initial rhythm, and AED and EMS defibrillator ECGs available, every additional minute of delay between emergency call and first shock was associated with a 6% lower proportion of successful defibrillation by the first shock. In patients who received the first shock within the first 2 minutes of the emergency call, ≈125 of 131 of the shocks (95%) were successful in terminating VF. After a delay to first shock of 4 to 5 minutes, successful termination of VF occurred in 434 of 466 (93%), and after a delay of >16 minutes, VF was successfully terminated in 111 of 148 (75%). A shorter delay to first shock was also associated with a higher proportion of organized rhythm after the first shock, a lower total number of shocks (indicating a lower incidence of VF recurrence), a higher proportion of transportation with sustained ROSC, and a higher rate of survival to discharge.

These findings provide further insights into the association between delay to first shock and survival.^4,8,18,20–22^ Early studies involving early deployment of AEDs in casinos or airports have shown significantly increased survival rates when delay to first shock was short.^22^ Studies on AED use by first responders, such as police, firefighters, and volunteer responders, have also shown the positive effects of early defibrillation on survival rates.^8,20^ Similar trends have been observed when bystanders use on-site AEDs to further reduce the delay to the first shock. Berdowski et al^18^ found that survival rates increased from 17% to 50% with the use of on-site AEDs. Alerting volunteer responders, directed to start basic life support or to fetch the nearest AED first, has proved to be an effective strategy to decrease the time to first shock and increase survival rate after OHCA with VF in residential areas.^4^

Whereas the relationship between shock delay and survival is well established and confirmed in our study, a substantial advancement of our study is that we could correlate delay to first shock with relevant immediate outcomes (ie, not only with termination of VF, but also with the rhythm immediately after the shock and the need for subsequent shocks). Reduction of the time to the first shock affects all components relevant for outcome after OHCA favorably: fewer unsuccessful shocks, less need for multiple shocks, and more return of organized rhythm after the first shock. This may partly explain the higher survival rate associated with a shorter delay to the first shock. In many epidemiological studies, the presence of VF as the first recorded rhythm has been considered an indicator of quality of the prehospital emergency response: a longer delay to arrival of EMS is associated with a lower probability of observing VF, more extensive cerebral damage, and a lower survival rate. This study shows that a rapid response has more beneficial effects than only observing VF more frequently and limiting the no-flow time.

The high proportion of successful shocks in VF of short duration can be attributed to multiple factors. Experimental studies in rabbit hearts have shown that VF characteristics, often analyzed by use of the amplitude spectrum area, become less favorable over time, and this corresponds to a decrease in VF termination rate.^23^ Similar observations have been reported in patients with OHCA, in whom a higher VF amplitude at the moment of defibrillation was strongly associated with successful defibrillation.^24^

On the cellular level, experimental studies have shown that a prolonged VF duration increases cardiac oxygen consumption and hampers restoration of the myocardial energy state and ventricular contractile function after simulated resuscitations.^25^ In a study using Langendorf-perfused porcine hearts in which VF was induced and maintained for 7 minutes, followed by simulated CPR in the presence or absence of VF, myocardial creatine–phosphate levels, which is a marker of myocardial energy state, were significantly higher in the absence of VF. Similarly, energy expenditure by electrical activity during cardiac ischemia accelerates the onset of irreversible myocardial damage.^26^ These findings support the importance of prompt termination of VF, also in case of recurrence, even in the presence of optimal CPR. This also explains why return of organized rhythm is observed frequently immediately after a first or later successful defibrillation shock, but ROSC is documented usually only after several minutes of continued CPR and replenishment of myocardial high-energy substrate.

It is a well-accepted fact that early defibrillation increases survival rate. This study helps clarify what “early” means and what its effects on outcomes are. There are several favorable factors associated with early defibrillation. First, a shock delivered quickly after the emergency call (eg, <6 minutes) was successful in 816 of 867 patients (94%), resulting in an organized rhythm in 335 of 867 patients (39%), absence of VF recurrence in 334 of 867 patients (39%), and 587 of 837 patients (70%) surviving to hospital discharge. In contrast, if delay to first shock was ≥10 minutes (a common situation), that shock was successful in 750 of 931 patients (81%), resulting in an organized rhythm in 207 of 931 patients (22%), absence of VF recurrence in 192 of 928 patients (21%), and 299 of 915 patients (33%) surviving to hospital discharge.

### Limitations

The main limitation was that the call to the dispatch center was used as a proxy for the time of initiation of VF in patients not witnessed by EMS. Thus, the true duration of VF until the first shock was longer. However, by including patients with a witnessed OHCA, the time interval between the true initiation of VF and the emergency call to the dispatch center was presumably very short in the majority of patients. Another limitation is the fact that the true initial rhythm is often unknown, as the rhythm might have changed before an AED or manual defibrillator was connected. Therefore, it might be possible that in some patients the cardiac arrest started with a pulseless VT that devolved into VF. In addition, some factors that can affect defibrillation success, such as chest compression quality and correct electrode pad placement, could not be determined by the current data collection.

### Conclusions

Every minute of delay to the first shock was associated with significantly lower proportions of VF termination, less return of organized rhythm, more VF recurrences, and less sustained ROSC during transport. This presumably explains the worse survival outcomes in patients with a long delay to defibrillation. Reducing the time interval from emergency call to first shock to ≤6 minutes could be considered a key performance indicator of the chain of survival, and interventions to meet this criterion should be initiated.

## ARTICLE INFORMATION

### Acknowledgments

The authors thank the data managers from ARREST, including Loes Bekkers, MSc, Paulien Homma, MSc, and Sandra van der Kroef-de Haas, MSc; Patrick Schober, MD, PhD, for expert opinion regarding the statistical methods; the collaborating dispatch centers, EMS services, first responders, and HartslagNu for cooperation and support; the students and EMS personnel who retrieved AED data; and Jerry Nolan, MD, for the suggestion that stimulated us to initiate this study.

### Sources of Funding

Dr Tan has received funding from the European Union Horizon 2020 research and innovation program under ESCAPE-NET (grant agreement 733381) and the COST (European Cooperation in Science and Technology) Action PARQ (grant agreement CA19137). Dr van Schuppen reports a grant from Stryker Emergency Care to his institution outside the scope of this study.

### Disclosures

None.

## Supplementary Material
